# Proteomic Expression Changes in Large Cerebral Arteries After Experimental Subarachnoid Hemorrhage in Rat Are Regulated by the MEK-ERK1/2 Pathway

**DOI:** 10.1007/s12031-017-0944-7

**Published:** 2017-07-24

**Authors:** Anne H. Müller, Alistair V.G. Edwards, Martin R. Larsen, Janne Nielsen, Karin Warfvinge, Gro K. Povlsen, Lars Edvinsson

**Affiliations:** 1 Department of Clinical Experimental Research, Glostrup Research Institute, Glostrup University Hospital, Nordre Ringvej 69, 2600 Glostrup, Denmark; 2 Department of Biochemistry and Molecular Biology, University of Southern Denmark, Campusvej 55, 5230 Odense M, Denmark; 3 Lund University, 223 62 Lund, Sweden

**Keywords:** Animal model, SAH, Proteomics, Mass spectrometry, MEK1/2 inhibition

## Abstract

Subarachnoid hemorrhage (SAH) is a serious clinical condition where leakage of blood into the subarachnoid space causes an acute rise in intracranial pressure and reduces cerebral blood flow, which may lead to delayed cerebral ischemia and poor outcome. In experimental SAH, we have previously shown that the outcome can be significantly improved by early inhibition of the MAPK/ERK kinase/extracellular signal-regulated kinase (MEK/ERK1/2) pathway. The aim of this study was to apply mass spectrometry to investigate the overall late effects of experimental SAH on cerebrovascular protein expression. SAH was induced in rats that were treated with the MEK1/2 inhibitor U0126 or vehicle. Neurological outcome was assessed using a battery of behavioral tests. Specific protein expression of large cerebral arteries was analyzed quantitatively with high-throughput tandem mass spectrometry. SAH resulted in a marked reduction of neurological scores, which was counteracted by U0126 treatment. Mass spectrometry analysis demonstrated regulation of 184 proteins after SAH, regulations that were in part prevented by U0126 treatment. Network analysis identified several protein networks including a strong structural network centered around 14-3-3. Additionally, protein networks with functions in mRNA metabolism and protein folding were identified. Treatment with U0126 inhibited cerebral vessel wall pERK1/2 expression and significantly improved outcome of the rats. In conclusion, we show that SAH induces a broad array of specific changes in the overall protein networks in cerebral artery smooth muscle cells and suggest that this is essential for understanding the vascular pathophysiology after SAH.

**Electronic supplementary material:**

The online version of this article (doi:10.1007/s12031-017-0944-7) contains supplementary material, which is available to authorized users.

## Introduction

Subarachnoid hemorrhage (SAH) is a condition caused by leakage of blood from a ruptured aneurysm into the subarachnoid space which causes an acute rise in intracranial pressure (ICP) and a diminished cerebral blood flow (CBF). During the acute phase, there is a mortality rate of about 15%. After cessation of the bleeding, patients might suffer from delayed cerebral ischemia (DCI) with pathological constriction of cerebral arteries, also known as cerebral vasospasm, in the days following the insult. The DCI phase is progressive and can result in infarction, neuronal loss, and poor outcome (Edvinsson and Povlsen 2011). Experimental studies have indicated plasticity of contractile cerebrovascular endothelin, 5-hydroxytryptamine 1B (5-HT_1B_), angiotensin AT_1_, and thromboxane A_2_ receptors in the ischemic process (Ansar and Edvinsson 2008; Ansar et al. 2010; Hansen-Schwartz et al. 2003). These receptor alterations are associated with reduced regional CBF and poor outcome (Ansari 2007).

Proteomics, blended from protein and genome, is large-scale studies of proteins. The possibility to obtain a picture of the total protein expression at a given time in a given tissue has greatly improved during the last 10 years with the accessibility of high-throughput mass spectrometry methods (Cottrell 2011). With this technology, thousands of proteins can be identified and quantified in a single study, which has made it possible to investigate, e.g., effects of diseases and treatments on protein expression in various tissues.

In experimental SAH in rats, we have previously shown that the outcome can be significantly improved by inhibition of the MAPK/ERK kinase/extracellular signal-regulated kinase (MEK-ERK) pathway days after the insult (Larsen et al. 2011). We reported that the MEK1/2 inhibitor U0126 prevents upregulation of the contractile receptors ET_B_ and 5-HT_1B_, and reduces vascular thickening evident 2 days post-SAH (Edvinsson and Povlsen 2011; Parker et al. 2013). Moreover, analysis of acute (0.5–1 h post insult) SAH in vivo events has shown SAH-induced regulation of focal adhesion molecules and actin cytoskeleton dynamics, and in addition a central role of activation of ERK1/2 (Parker et al. 2013).s

The detailed analysis of acute SAH events by Parker et al. (Parker et al. 2013) revealed the proteomic changes immediately associated with the insult. However, no large-scale proteomic analysis of the alterations in proteins 48 h after SAH, a time-point more closely related to the development of DCI, has yet to be performed. Hence, the aim of this study was to apply high-throughput quantitative tandem mass spectrometry to establish later effects of experimental SAH on the protein expression in the cerebral vessels. Moreover, because the inhibition of MEK-ERK1/2 activation has a prophylactic effect in later cerebrovascular changes after SAH, we investigated the effects of U0126 on the SAH-induced proteomic changes (Larsen et al. 2011).

## Experimental Procedures

All animal procedures were carried out strictly within national laws and guidelines and were approved by the Danish Animal Experimentation Inspectorate (license no. 2011/561-2025).

### Rat Subarachnoid Hemorrhage Model

SAH was induced as described by Prunell et al. (2002). Male Sprague-Dawley rats 350–400 g (Taconic, Denmark) were anesthetized using 3.5% isoflurane (Baxter A/S, Denmark) in atmospheric air and O_2_ (70:30). Anesthesia was maintained by intubation and artificial ventilation of 1–2% isoflurane in N_2_O/O_2_ (70:30) during the surgical procedure. Respiration was monitored by regularly withdrawing blood samples to a blood gas analyzer (Radiometer, Denmark). A temperature probe was rectally inserted to record the body temperature, which was maintained at 37 °C by a heating pad. ICP was measured via a catheter inserted into the basal cisterna via a hole in the atlanto-occipital membrane. The catheter was connected to a pressure transducer and the signal was recorded in the software LabChart via a PowerLab (both from AD Instruments, UK). Mean arterial blood pressure (MABP) was measured via a tail artery catheter connected to a pressure transducer and recorded in LabChart. Cortical CBF was measured with a laser-Doppler fiber optic probe placed directly on the dura mater on the surface of the brain via a hole drilled through the skull, 4 mm anterior from the bregma and 3 mm to the right of the midline, without perforation of the dura. ICP, MABP, and CBF were measured in real time with recordings commencing approximately 30 min before SAH and continuing until 1 h after SAH.

A 27G blunt cannula was stereotactically placed 6.5 mm anterior to the bregma in the midline at an angle of 30° to the vertical plane, it was lowered until it met the base of the scull, and then retracted 1 mm placing the tip of the needle just in front of the chiasma opticum. Rats equilibrated 30 min before 300 μl of blood was withdrawn from the tail catheter and injected manually into the prechiasmatic cisterna at a pressure equal to the MABP (80–100 mmHg). Rats were maintained under anesthesia for another 60 min in order to recover. The ICP catheter was cut and sealed with a removable plug 2 cm from the tip. The tail catheter, the needle, and the laser-Doppler probe were removed and incisions closed. Carprofen (4 mg/kg; Pfizer, Denmark) against pain was administered subcutaneous as well as 15 ml saline for hydration. Rats were then revitalized and extubated. Sham-operated rats went through the same procedure, with the exception that no blood was injected intracisternally.

### Experimental Groups

A total of 40 rats underwent surgery for the neurological evaluation and mass spectrometric analysis and 18 rats underwent surgery for Western blot validation. The mass spectrometry was conducted in duplicates and each sample consisted of tissue from four to five rats. These rats also went through neurological assessment, meaning that there were 9–10 biological replicates in each group for neurological evaluation. Western blot was carried out in technical duplicates with four to six biological replicates and each sample consisted of protein purified from one rat. In order to assess whether MEK-ERK1/2 activity was involved in the vascular response in the first 2 days post-SAH, thus, we compared animals treated with either U0126 (1,4-diamino-2,3-dicyano-1,4-bis [2-aminophenylthio] butadiene) (Duncia et al. 1998) or vehicle (DMSO) at 6, 12, 24, and 36 h post-SAH with sham-operated rats that received neither U0126 nor vehicle.

Animals were randomly selected for treatment with either U0126 or vehicle. U0126 was given as 0.05 ml/kg body weight of a 10^−5^ M solution of U0126 ethanolate (Sigma-Aldrich, MO, USA) in isotonic saline with 0.1% DMSO, yielding a final dose of 0.22 μg /kg body weight. Vehicle consisted of 0.1% DMSO in isotonic saline. Treatment was administered intracisternal through the ICP catheter placed with the tip in the basal cistern.

At 48 h post-surgery, all rats were anesthetized using CO_2_ and decapitated. In a dissection microscope, the middle cerebral arteries (MCAs), the circle of Willis, and the basilar artery were carefully dissected free of brain tissue and cleaned of connective tissue and blood, and stored at −80 °C.

### Neurological Function

Neurological evaluations (rotating pole test and spontaneous behavior observations) were performed by personnel blinded with regard to experimental groups. Tests and observations were performed in a silent room with as few visual disturbing elements as possible.

#### Rotating Pole Test

Gross sensorimotor function of animals was evaluated by testing their balance and coordination of movements on a rotating pole (45 mm in diameter and 150 cm in length), which was either steady or rotating at different speeds (3 or 10 rpm) (Ohlsson and Johansson 1995). A cage where the floor was covered with bedding material from the home cage of the rat was placed at the end of the pole opposite from the rat to serve as a positive reinforcement for the rat to traverse the pole, and the ability of the rats to traverse the pole was monitored. The performance of the rat was scored according to the following definitions: Score 1, the animal is unable to sit on the pole and falls off immediately. Score 2, the animal has severe difficulties to stay on the pole, but manages to balance for a while and move less than 30 cm on the pole. Score 3, the animal embraces the pole with paws while crossing, but manages to move more than 30 cm. Score 4, the animal manages to traverse the pole and reach the platform but moves with the body close to the pole, embraces the pole, or jumps with hind legs. Score 5, the animal traverse the pole with normal posture but displays one or several of the following deficits: foot slips (less than 10), slightly disturbed pattern of movement, stops along the pole, and has difficulty staying on the pole while standing still. Score 6, the animal traverses the pole perfectly with 0–2 ft slips and no stops. Results were analyzed with one-way ANOVA with Dunn’s multiple comparison post-test. Significance level was set to *p* < 0.05.

#### Spontaneous Behavior

Spontaneous activity of the rats was quantified by placing the rats individually in a test cage with fresh bedding and nesting material for 20 min. An observer equipped with a timer recorded all time intervals spent moving around in the cage (locomotion), sitting or lying in the same place (no movement), rearing, or grooming. Results were analyzed with one-way ANOVA and Newman-Keuls multiple comparison post-test. Significance level was set to *p* < 0.05.

### Protein Purification

Tissue from cerebral arteries was dounce homogenized in ice-cold 0.1 M Na_2_CO_3_ containing 1 tablet PhosSTOP phosphatase inhibitor cocktail (Roche, France) per 10 ml and 1 ml P8340 protease inhibitor (Sigma-Aldrich, MO, USA) per 20 ml, and then tip-probe sonicated 2 × 20 s on ice. Homogenates were ultra-centrifuged at 150,000×*g* for 1.5 h. Pellets (membrane fractions) were resuspended in 6 M urea/2 M thiourea. Supernatants (soluble fractions) were precipitated using 20% trichloroacetic acid, and pellets from this precipitation were resuspended in 6 M urea/2 M thiourea. Samples were reduced in 10 mM dithiothreitol, then alkylated using 20 mM iodoacetamide. Lysyl-endopeptidase (Wako, Osaka, Japan) was added for 3 h, and then samples were diluted 6-fold in 50 mM triethyl ammonium bicarbonate. Trypsin (Promega, WI, USA) was added at a ratio of 1:40 (*w*/*w*) and left for 18 h to digest. Digests were acidified by addition of formic acid to a final concentration of 2% and the protein contents of samples were quantified using amino acid composition analysis. Equal amounts of protein from each sample were iTRAQ four-plex labeled according to the manufacturer’s instructions (AB Sciex, MA, USA), equal labeling was confirmed on a Bruker UltraFlex MALDI MS/MS instrument (Bruker, MA, USA), and samples were combined to give equal reporter intensity**.**

Combined peptide samples were cleaned up on a homemade R3 column and then dried down for hydrophilic interaction liquid chromatography (HILIC). Peptide samples were resuspended in 90% acetonitrile (ACN)/0.1% trifluoroacedic acid (TFA) and loaded onto a micro-HILIC HPLC chromatography system. The HILIC resin was TSK-gel Amide 80 (Tosoh Bioscience, Japan). Samples were separated over a gradient of 90–60% organic solvent over 30 min, then 60–0% over 15 min. Fractions were collected every minute at absorbances over 500 mAU and every 5 min otherwise. All fractions were lyophilized.

### Mass Spectrometry

Dry peptide fractions were resuspended in 0.1% FA and loaded onto a Thermo Easy-LC system. Peptides were eluted using a 0–34% organic gradient over 70 min, then 34–100% over 20 min. All LC-MS runs were performed on columns with a 75-μm inner diameter, packed with C18 material (Dr. Maisch, Ammerbuch-Entringen, Germany). Mass spectrometry (MS) was performed using higher-energy collisional dissociation (HCD) fragmentation on a Thermo LTQ Velos (Thermo Fisher Scientific, Germany). Quantification was performed on reporter tags at *m*/*z* 114, 115, 116, and 117. MS was performed with the following settings: A full MS scan in the mass area of 400–1800 Da was performed in the Orbitrap with a resolution of 30,000 FWHM and a target value of 1 × 10^6^ ions. For each full scan, the seven most intense ions (>+1 charge state) were selected for HCD and detected at a resolution of 7500 FWHM. HCD was performed with the following settings: threshold for ion selection was 20,000, the target value of ions used for HCD was 1 × 10^5^, and activation time was 1 ms, the isolation window was 2.5 Da, and the normalized collision energy was 48.

### Analysis of Mass Spectrometry Data

Data was searched using Thermo Proteome Discoverer (version 1.3.0.339) and Mascot (v2.2, Matrix Science Ltd., UK) allowing for variable methionine oxidation. Enzyme specificity was set for trypsin, with two missed cleavages allowed. Precursor mass tolerance was 10 ppm and fragment mass tolerance was set to 0.05 Da. iTRAQ labeling was also set as a variable modification in order to detect lysine modification, while cysteine carbamidomethylation was set as a fixed modification. Data were searched against a Swiss-prot rodent database (UniProtKB/Swiss-Prot 2012_10). Data were filtered to 1% peptide FDR with a decoy approach using percolator and filtered to remove Mascot scores less than 18, as previously described (Engholm-Keller et al. 2012). Data was exported from Proteome Discoverer and manually normalized to protein median values across an entire labeling experiment to correct for protein abundance variation. Protein and peptide regulation was defined based on the log2-transformed iTRAQ ratios and considered regulated when exceeding 2 standard deviations from the median ratio for a given tag. Proteins were classified according to their molecular function using the PANTHER database (www.panther.db) and network analysis was performed using STRING, version 9 (www.string.db) (Szklarczyk et al. 2011).

### Western Blot

Each purified protein sample was prepared from MCAs, circle of Willis, and the basilar artery from one animal. Samples were sonicated in RIPA buffer (50 mM Tris-HCl buffer pH 7.5, 150 mM NaCl, 1 mM EDTA pH 8.0, 1% NP-40, 0.1% SDS, 0.5% Triton X-100, 50 mM β-glycerolphosphate, 0.1% deoxycholic acid) with 1 tablet Complete Protease Inhibitor Cocktail (Roche, France) per 50 ml, and 1 tablet PhosSTOP Phosphatase Inhibitor Cocktail (Roche, France) per 10 ml RIPA.

Protein concentrations were determined with a spectrophotometer (Infinite M200, TECAN, Switzerland). Purified protein samples were run on a 4–20% precast SDS gel (Expedeon Inc., USA) and blotted onto a nitrocellulose membrane (GE Healthcare, USA). Membranes were blocked for 1 h in 5% bovine serum albumin (BSA) in Tris-buffered saline (50 mM Tris, 150 mM NaCl, pH 7.6) with 0.05% (*v*/*v*) Tween-20 (TBS-T) at room temperature and incubated over night at 4 °C with primary antibodies diluted in 5% BSA TBS-T (Supplementary Table S1). After a 5 min wash, the membranes were incubated 1 h at room temperature with secondary antibody diluted in 5% BSA TBS-T (Supplementary Table S1), washed five times 5 min in TBS-T and developed using ECL Select (GE Healthcare, USA) and a LAS4000 (Fujifilm, Japan). Blots were quantified with Image Gauge V3.2 (Fujifilm, Japan) and intensities for band representing 14-3-3 were normalized to intensities for bands representing SM22 which was used as loading control. Normalized intensities for the U0126-treatment and DMSO-treatment groups, respectively, were compared to normalized intensities for the sham-surgery group using one-way ANOVA and Newman-Keuls test for multiple comparison significance; the level was set to *p* < 0.05.

### Immunohistochemistry

Immunohistochemical staining was performed on cerebral vessel segments (from three rats of the sham group, SAH with vehicle or U0126 group). The tissues were embedded in Tissue-Tek®, frozen on dry ice, and stored at −80 °C until further processing. They were sectioned (10 μm) on a cryostat (Leica, Denmark) and mounted on microscope slides (SuperFrost®, Menzel, Germany). The sections were fixed for 20 min using Stefanini’s fixative (2% paraformaldehyde and 0.2% picric acid in phosphate buffer, pH 7.2) and permeabilized in phosphate-buffered saline (PBS) containing 0.25% Triton X-100 (T-PBS). To prevent nonspecific staining, sections were blocked with 2% donkey serum and 1% BSA in T-PBS for 1 h. Samples were incubated at 4 °C overnight with primary anti-pERK1/2 antibody (1:250, alx-210-506a, Alexis Biochemicals, Nottingham, UK) diluted in T-PBS. The following day, the slides were rinsed three times in T-PBS and incubated with secondary donkey anti-sheep DyLight™ 488 antibody (1:200, 713-485-003, Jackson ImmunoResearch, UK) or secondary donkey anti-mouse FITC (1:100, Jackson Immunoresearch Laboratories) for 1 h in room temperature followed by three washes in T-PBS before mounting with Vectashield (Vector Laboratories, Burlingame, CA, USA). Primary antibody was omitted as a negative control.

## Results

### Physiological Parameters

Two groups of animals were subjected to SAH and then treated with either U0126 or vehicle at 6, 12, 24, and 36 h post-SAH. A third group of animals were subjected to sham surgery and received neither U0126 nor vehicle (Supplementary Fig. S1).

Measurements of the physiological parameters during SAH surgery showed that ICP reached values of 10–20 mmHg above MABP (80–100 mmHg) and cortical CBF was lowered to 75% compared to flow before insult, and did not return to baseline for a minimum of 10 min. These results (Supplementary Table S2) are comparable with values in our previous study (Povlsen et al. 2013) and values did not differ between groups. The mortality rate after 48 h was 16%.

### Neurological Functions

**Fig. 1:**
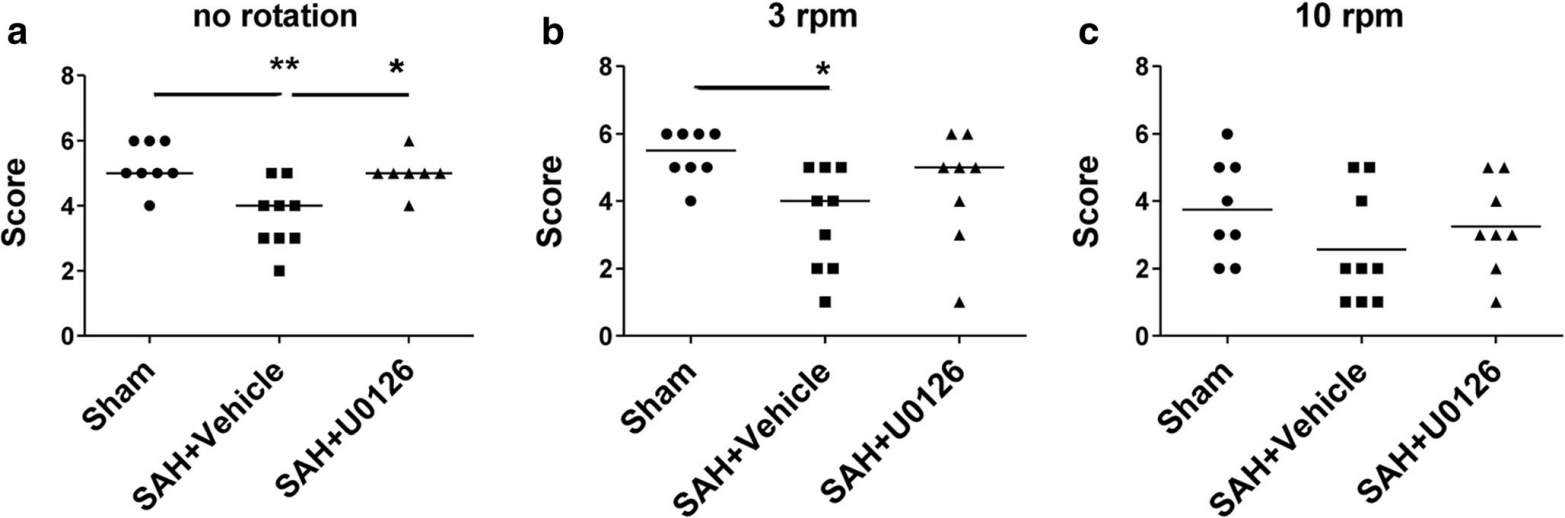
Neurological assessment of rats treated with U0126 or DMSO post-SAH. Rats were tested for their ability to traverse a 1.5-m pole with either no rotation, 3, or 10 rpm. Each data point is the score of one rat; *n* = 7–9. *Horizontal lines* are median score. Compared with sham-operated animals, rats that underwent SAH performed significantly poorer at no rotation (**a**) and 3 rpm (**b**). At 10 rpm (**c**), there was no significant difference between sham and SAH. Animals that underwent SAH and were treated with U0126 performed significantly better than animals treated with vehicle at no rotation (**a**). Data were analyzed with one-way ANOVA and Dunn’s post-test: * = *p* < 0.05, ** = *p* < 0.01

Neurological function was assessed using the rotating pole test and standardized observations of spontaneous behavior by trained personnel blinded to the experimental groups. The ability of the rats to traverse a rotating pole at either no rotation, at 3, or 10 rpm was significantly decreased by induction of SAH. This reduction was restored by U0126 treatment (Fig. 1). The administration of U0126 in SAH animals was well tolerated and did not modify ICP, CBF, or MABP differently as compared to the DMSO-treated SAH group (Larsen et al. 2011).

**Fig. 2:**
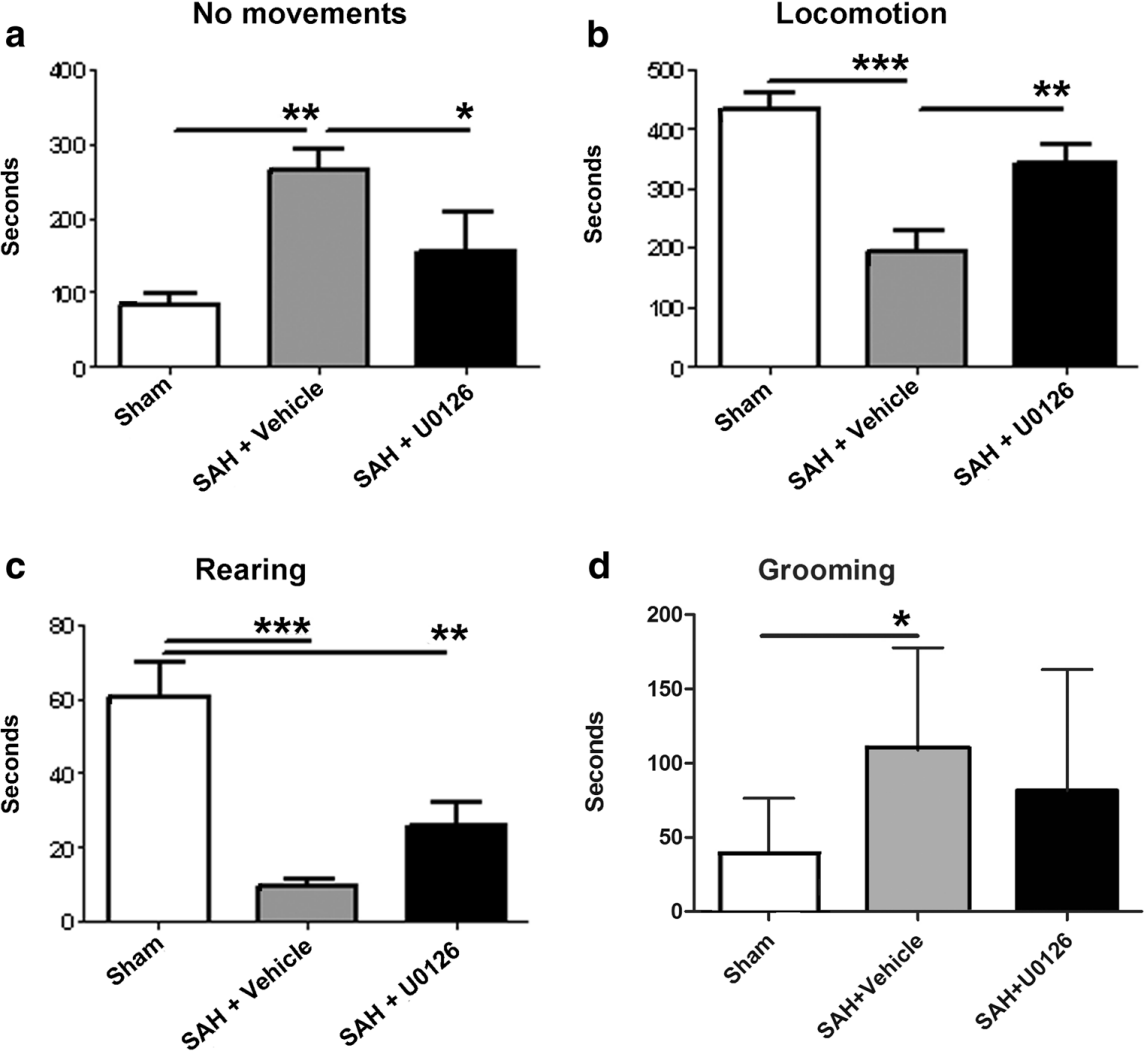
Behavioral observations of rats treated with U0126 or vehicle post-SAH. Rats were observed for 20 min and the duration of their spontaneous activities was quantified. Data are mean time spent on each activity ± S.E.M.; *n* = 8–9. Compared with sham-operated animals, rats that underwent SAH performed significantly poorer with no movement (**a**), locomotion (**b**), rearing (**c**), and grooming (**d**). Animals that underwent SAH and were treated with U0126 performed significantly better than animals treated with vehicle with no movement (**a**) and locomotion (**b**). Data were analyzed with one-way ANOVA and Newman-Keuls post-test for multiple comparison: * = *p* < 0.05, ** = *p* < 0.01, *** = *p* < 0.001

Observations on time spend with no movements, locomotion, rearing, and grooming were recorded using a standardized protocol. It was found that induction of SAH worsened the neurological function of rats as seen by a significant change in time spent with no movement and locomotion (Fig. 2). This change was significantly neutralized by U0126 treatment. In addition, SAH markedly decreased time spent with rearing (*p* < 0.001) and increased the time of grooming; these effects were partly restored after U0126 treatment (*p* < 0.05) (Fig. 2).

In conclusion, inhibition of vessel wall pERK1/2 (U0126 treatment) of rats subjected to SAH improved the neurology functional parameters as compared to treatment with vehicle.

### Proteome Analysis in SAH in the Absence or Presence of MEK1/2 Inhibition

In order to obtain an overview of protein expression in cerebral vessels after induction of SAH and to assess the effect of treatment with the MEK1/2 inhibitor U0126, tandem mass spectrometry with isobaric tags for relative and absolute quantitation (iTRAQ) was used to generate quantitative total proteomes for the three groups of animals (sham, vehicle-, or U0126 treatment) (Ong and Mann 2005; Ross et al. 2004; Steen and Mann 2004).

The analysis of the cerebral vessel proteome was performed in duplicates, each sample containing a pool of cerebral vessels (MCAs, circle of Willis, and basilar artery) from four to five animals dissected 48 h after rats were subjected to either SAH (and treated with either U0126 or vehicle) or sham surgery. Pilot studies showed no effect of vehicle per se or of U0126 to non-SAH sham surgery animals (data not shown).

The cerebral artery samples were iTRAQ labeled as follows: U0126-treated groups were labeled with iTRAQ 114, vehicle-treated groups were labeled with iTRAQ 115, and sham groups were labeled with iTRAQ 116. Tandem mass spectrometry was performed on a 1:1:1 mixture of all three groups simultaneously. Protein levels in the different groups were determined based on the intensities of the tags compared with sham. Because this was an overall screening proteome study, the results include all identified proteins in the groups.

#### Core Results

A total of 2677 proteins were identified. The effect of SAH on protein expression in cerebral vessels (without any contamination of brain tissue) was assessed by comparing the regulated proteins of rats subjected to SAH and treated with vehicle with the regulated proteins of rats subjected to sham surgery (this group did not differ in expression from sham surgery with or without U0126; data not shown). When examining the SAH vehicle group, we identified 171 upregulated proteins and 79 downregulated proteins compared with sham. The ten most upregulated and downregulated proteins are shown in Table 1A and B. Using the PANTHER database, we sorted the proteins based on their molecular function.^1^ Of the 171 upregulated proteins, 159 were recognized in the database with a total of 171 hits.^2^ Of the allocated proteins, 39.9% (68 proteins) were involved in catalytic activity, 30.4% (52 proteins) in binding of either nucleic acids, proteins, or calcium ions, 17% (29 proteins) in structural activity, 1.8% (3 proteins) in enzyme regulator activity, 2.9% (5 proteins) in transcription factor activity, 3.5% (6 proteins) in receptor activity, 12.3% (4 proteins) in translation, and 2.3% (4 proteins) have transporter functions (Supplementary Fig. S2).

**Table 1:** The ten most upregulated and downregulated proteins after SAH. The table shows proteins that are regulated after SAH and the level of regulation is expressed as log_2_ of the iTRAQ ratios. (A) The ten most upregulated proteins after SAH when compared with sham. (B) The ten most downregulated proteins after SAH when compared with sham

Accession number	Protein name	Log_2_
A: The ten most upregulated proteins after SAH		
P50116	Protein S100-A9	3.96
9JJ54	Heterogeneous nuclear ribonucleoprotein D0	3.84
Q9Z1Z3	Epsin-2	3.78
Q925G0	Putative RNA-binding protein 3	3.74
B3EWD2	Hemoglobin subunit beta	3.70
Q8VC52	RNA-binding protein with multiple splicing 2	3.70
Q9DBR1	5′-3′ Exoribonuclease 2	3.58
Q63083	Nucleobindin-1	3.58
P11348	Dihydropteridine reductase	3.57
P63281	SUMO-conjugating enzyme UBC9	3.55
B: The ten most downregulated proteins after SAH		
Q62715	Neutrophil antibiotic peptide NP-2	−5.38
P04764	Alpha-enolase	−5.38
D3ZLY9	Histone H2B type 1-H	−4.82
P05811	Alpha-crystallin B chain	−4.47
Q5RKG9	Eukaryotic translation initiation factor 4B	−4.09
Q6LED0	Histone H3.1	−3.96
P04646	60S ribosomal protein L35a	−3.85
P08081	Clathrin light chain A	−3.75
P08009	Glutathione S-transferase Yb-3	−3.64
Q9JM53	Apoptosis-inducing factor 1, mitochondrial	−3.42

Of the 79 downregulated proteins, 72 were found in the PANTHER database and gave 82 hits. Of these proteins, 30.5% (25 proteins) were involved in binding,^3^ 29.3% (24 proteins) in catalytic activity, 15.3% (11 proteins) in structural activity, 9.8% (8 proteins) have transporter functions, 7.3% (6 proteins) are involved in receptor activity, 6.1% (5 proteins) in enzyme regulator activity, 2.4% (2 proteins) in translation, and 1.2% (1 protein) in transcription factor activity (Supplementary Fig. S2). This demonstrates that the response to SAH in the major cerebral arteries involves both up- and downregulation of mainly proteins with binding, catalytic, and structural functions. The stronger of the two responses seems to be upregulation of proteins rather than downregulation. Importantly, we show that a wide range of proteins were regulated after SAH, hence targeting a single protein is not a viable way to counteract the detrimental effects of SAH.

### The Effect of MEK1/2 Inhibition

**Fig. 3:**
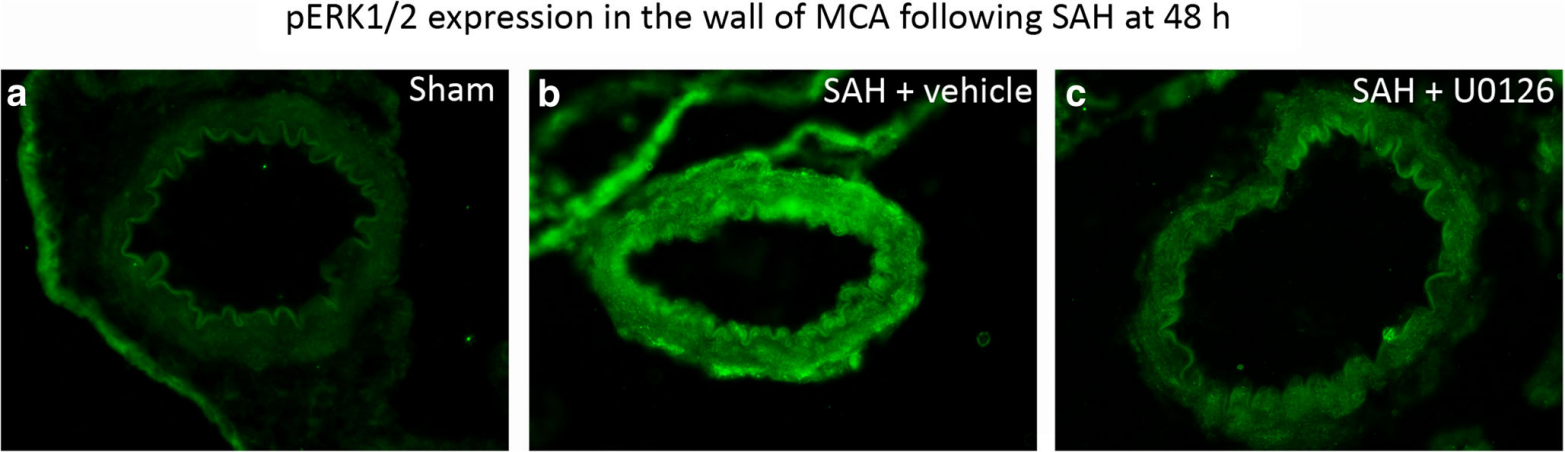
pERK immunohistochemistry on middle cerebral artery. **a** The image shows MCA of a sham-operated animal. No pERK is visible in the vessel. **b** The image demonstrates specific pERK immunoreactivity in the vessel after induction of SAH. **c** Treatment with U0126 attenuates the pERK immunoreactivity in the vessel

The first part was verification in the circle of Willis arteries that treatment with U0126 had efficacy per se. This has been done in a Western blot quantitative study before using the same SAH method (Ansar and Edvinsson 2008). Here, we confirm this effect using immunohistochemistry. We verified that SAH induced enhanced expression of pERK1/2 in the cerebral vascular smooth muscle cells (VSMC), while this was unaltered in the U0126-treated vessels (Fig. 3). The effect of MEK1/2 inhibition on SAH-induced changes in protein expression was assessed in a two-step procedure where the first step was to compare SAH animals treated with U0126 with sham animals. Hereby, 104 proteins were identified as upregulated and 183 proteins were found to be downregulated, which indicates that MEK1/2 inhibition affects the SAH-induced expressional changes in cerebral vessels. The next step in order to identify downstream targets of MEK1/2 was to search for proteins that fulfilled one of the following criteria: 1, the protein is upregulated in vehicle-treated animals (as compared with sham animals) and either downregulated or not affected in U0126-treated animals (as compared with sham animals). 2, The protein is downregulated in vehicle-treated animals (as compared with sham animals) and either upregulated or not affected in U0126-treated animals (as compared with sham animals). A list of 133 proteins (Table 2) was identified using criteria 1. This group (hereafter denoted group 1) represents proteins with an SAH-induced expressional increase that does not persist after MEK1/2 inhibition. A list of 51 proteins (Table 3) was identified based on criteria 2. This group (hereafter denoted group 2) represents proteins with an SAH-induced expressional decrease that does not persist after MEK1/2 inhibition.

**Table 2:** Proteins that show a SAH-induced upregulation which does not persist after MEK inhibition. The list shows proteins that fulfilled the following criteria: the protein is upregulated in vehicle-treated animals (as compared with sham animals) and either downregulated or not affected in U0126-treated animals (as compared with sham animals). Regulation is expressed as log_2_ of the iTRAQ ratios. This group of 133 proteins is referred to as group 1 and represents proteins with an SAH-induced expressional increase that does not persist after MEK1/2 inhibition

Accession number	Protein name	Log_2_
Q9JJ54	Heterogeneous nuclear ribonucleoprotein D0	3.84
Q4FZY0	EF-hand domain-containing protein D2	3.78
Q9Z1Z3	Epsin-2	3.78
Q925G0	Putative RNA-binding protein 3	3.74
B3EWD2	Hemoglobin subunit beta	3.70
B5DFF2	RNA-binding protein with multiple splicing 2	3.70
Q9DBR1	5′-3′ Exoribonuclease 2	3.58
Q63083	Nucleobindin-1	3.58
P11348	Dihydropteridine reductase	3.57
P63281	SUMO-conjugating enzyme UBC9	3.55
Q8BQ30	Phostensin	3.54
Q6P686	Osteoclast-stimulating factor 1	3.52
Q63797	Proteasome activator complex subunit 1	3.46
Q8K297	Procollagen galactosyltransferase 1	3.45
Q4KM73	UMP-CMP kinase	3.41
Q924S5	Lon protease homolog, mitochondrial	3.38
Q9QX47	Protein SON	3.34
Q5U318	Astrocytic phosphoprotein PEA-15	3.33
P61149	Fibroblast growth factor 1	3.28
Q5BJK8	Golgi integral membrane protein 4	3.28
O08586	Phosphatidylinositol-3,4,5-trisphosphate 3-phosphatase	3.25
D3ZQN7	Laminin subunit beta-1	3.22
P62815	V-type proton ATPase subunit B, brain isoform	3.22
Q9DBZ5	Eukaryotic translation initiation factor 3 subunit K	3.16
P61972	Nuclear transport factor 2	3.15
Q4FZY0	EF-hand domain-containing protein D2	3.13
Q6PEV3	WAS/WASL-interacting protein family member 2	3.11
P63326	40S ribosomal protein S10	3.10
Q6AY09	Heterogeneous nuclear ribonucleoprotein H2	3.03
Q63663	Interferon-induced guanylate-binding protein 2	3.02
P52303	AP-1 complex subunit beta-1	2.97
P60868	40S ribosomal protein S20	2.97
Q64611	Cysteine sulfinic acid decarboxylase	2.90
P51583	Multifunctional protein ADE2	2.89
Q63871	DNA-directed RNA polymerases I, II, and III subunit RPABC4	2.89
Q5U211	Sorting nexin-3	2.87
P27615	Lysosome membrane protein 2	2.86
Q4KM65	Cleavage and polyadenylation specificity factor subunit 6	2.86
Q62865	cGMP-inhibited 3′,5′-cyclic phosphodiesterase A	2.85
P55260	Annexin A4	2.84
Q8R361	Rab11 family-interacting protein 5	2.81
P97379	Ras GTPase-activating protein-binding protein 2	2.81
Q66H20	Polypyrimidine tract-binding protein 2	2.78
Q6AXS3	Protein DEK	2.72
P36876	Serine/threonine-protein phosphatase 2A 55 kDa regulatory subunit B alpha isoform	2.72
B5DFC8	Eukaryotic translation initiation factor 3 subunit C	2.72
Q9QWE9	Gamma-glutamyltransferase 5	2.71
Q63433	Serine/threonine-protein kinase N1	2.70
Q4KM74	Vesicle-trafficking protein SEC22b	2.69
Q5U2T8	Corepressor interacting with RBPJ 1	2.64
Q9WVA3	Mitotic checkpoint protein BUB3	2.61
Q8CIE6	Coatomer subunit alpha	2.59
G3V9C7	Histone H2B type 1-K	2.57
Q9Z340	Partitioning defective 3 homolog	2.55
P97633	Casein kinase I isoform alpha	2.47
P41499	Tyrosine-protein phosphatase non-receptor type 11	2.46
P60711	Actin, cytoplasmic 1	2.45
Q9R1Z8	Vinexin	2.45
Q63560	Microtubule-associated protein 6	2.42
Q27W02	Protein mago nashi homolog	2.39
Q6AYD6	PDZ and LIM domain protein 2	2.37
Q4AEF8	Coatomer subunit gamma	2.37
P52481	Adenylyl cyclase-associated protein 2	2.37
P49138	MAP kinase-activated protein kinase 2	2.36
Q99P96	Histone deacetylase 7	2.36
P36372	Antigen peptide transporter 2	2.35
P05811	Alpha-crystallin B chain	2.34
Q5HZV9	Protein phosphatase 1 regulatory subunit 7	2.33
P70615	Lamin-B1	2.32
Q9WUX5	Protein MRVI1	2.32
P16675	Lysosomal protective protein	2.31
Q9EPH8	Polyadenylate-binding protein 1	2.29
Q63560	Microtubule-associated protein 6	2.28
Q60436	Serrate RNA effector molecule homolog (Fragment)	2.26
Q8R081	Heterogeneous nuclear ribonucleoprotein L	2.26
Q561S0	NADH dehydrogenase [ubiquinone] 1 alpha subcomplex subunit 10, mitochondrial	2.26
Q5U2R0	Methionine adenosyltransferase 2 subunit beta	2.24
P46664	Adenylosuccinate synthetase isozyme 2	2.21
P0C5W1	Microtubule-associated protein 1S	2.19
Q9WTT6	Guanine deaminase	2.17
O55125	Protein NipSnap homolog 1	2.17
Q6MG49	Large proline-rich protein BAG6	2.16
P04692	Tropomyosin alpha-1 chain	2.16
Q8VC85	U6 snRNA-associated Sm-like protein LSm1	2.16
Q6AYE2	Endophilin-B1	2.16
D3ZU13	Eukaryotic translation initiation factor 4 gamma 1	2.15
Q6MG61	Chloride intracellular channel protein 1	2.14
O55012	Phosphatidylinositol-binding clathrin assembly protein	2.13
Q5U301	A-kinase anchor protein 2	2.13
Q9Z2K1	Keratin, type I cytoskeletal 16	2.13
Q9EPL8	Importin-7	2.10
P14426	H-2 class I histocompatibility antigen, D-K alpha chain	2.06
Q8BSY0	Aspartyl/asparaginyl beta-hydroxylase	2.05
Q6AYQ4	Transmembrane protein 109	2.02
O55096	Dipeptidyl peptidase 3	2.01
P39069	Adenylate kinase isoenzyme 1 (Fragments)	1.98
P05982	NAD(P)H dehydrogenase [quinone] 1	1.34
Q9QXQ0	Alpha-actinin-4	1.27
Q62507	Cochlin	1.26
P19132	Ferritin heavy chain	1.20
P38983	40S ribosomal protein SA	1.17
P52944	PDZ and LIM domain protein 1	1.17
P70619	Glutathione reductase (Fragment)	1.14
P24368	Peptidyl-prolyl cis-trans isomerase B	1.12
P38652	Phosphoglucomutase-1	1.07
P63326	40S ribosomal protein S10	1.06
Q5XI28	Ribonucleoprotein PTB-binding 1	1.05
Q9Z1P2	Alpha-actinin-1	1.05
B0BNF1	Septin-8	1.04
P97633	Casein kinase I isoform alpha	1.04
P63102	14-3-3 protein zeta/delta	1.03
Q60972	Histone-binding protein RBBP4	0.99
Q9DB16	Calcium-binding protein 39-like	0.96
Q497B0	Omega-amidase NIT2	0.96
B0BNA7	Eukaryotic translation initiation factor 3 subunit I	0.95
P47853	Biglycan	0.94
Q921M3	Splicing factor 3B subunit 3	0.93
O55126	Protein NipSnap homolog 2	0.93
Q8VHI3	GDP-fucose protein O-fucosyltransferase 2	0.92
P61983	14-3-3 protein gamma	0.91
P28661	Septin-4	0.89
P14046	Alpha-1-inhibitor 3	0.88
P15651	Short-chain specific acyl-CoA dehydrogenase, mitochondrial	0.87
P19804	Nucleoside diphosphate kinase B	0.86
P85972	Vinculin	0.85
P15865	Histone H1.2	0.84
Q58FK9	Kynurenine--oxoglutarate transaminase 3	0.84
P62260	14-3-3 protein epsilon	0.84
Q5XI78	2-Oxoglutarate dehydrogenase, mitochondrial	0.82
B2GV06	Succinyl-CoA:3-ketoacid-coenzyme A transferase 1, mitochondrial	0.82
P31977	Ezrin	0.81
Q01129	Decorin	0.80
O88483	[Pyruvate dehydrogenase [acetyl-transferring]]-phosphatase 1, mitochondrial	0.80
P18418	Calreticulin	0.80
P12007	Isovaleryl-CoA dehydrogenase, mitochondrial	0.79
O35854	Branched-chain-amino-acid aminotransferase, mitochondrial	0.78
Q66HS7	PDZ and LIM domain protein 3	0.78
P07335	Creatine kinase B-type	0.77
Q9ESW0	DNA damage-binding protein 1	0.77

**Table 3:** Proteins that show a SAH-induced downregulation which does not persist after MEK inhibition. The list shows proteins that are downregulated in vehicle-treated animals (as compared with sham animals) and either upregulated or not affected in U0126-treated animals (as compared with sham animals). Regulation is expressed as log_2_ of the iTRAQ ratios. This group of 51 proteins is denoted group 2 and represents proteins with an SAH-induced expressional decrease that does not persist after MEK1/2 inhibition

Accession number	Protein name	Log_2_
Q64478	Histone H2B type 1-H	−4.82
P05811	Alpha-crystallin B chain	−4.47
Q8BGD9	Eukaryotic translation initiation factor 4B	−4.09
P08009	Glutathione S-transferase Yb-3	−3.64
Q5U2R0	Methionine adenosyltransferase 2 subunit beta	−3.39
P62860	40S ribosomal protein S30	−3.29
P23457	3-Alpha-hydroxysteroid dehydrogenase	−3.25
Q9Z2G8	Nucleosome assembly protein 1-like 1	−3.14
P55063	Heat shock 70 kDa protein 1-like	−3.03
Q8VDM6	Heterogeneous nuclear ribonucleoprotein U-like protein 1	−2.93
P22057	Prostaglandin-H2 D-isomerase	−2.87
P04897	Guanine nucleotide-binding protein G(i) subunit alpha-2	−2.76
P27321	Calpastatin	−2.76
Q04940	Neurogranin	−2.72
O35206	Collagen alpha-1(XV) chain	−2.72
Q9CSU0	Regulation of nuclear pre-mRNA domain-containing protein 1B	−2.39
Q63362	NADH dehydrogenase [ubiquinone] 1 alpha subcomplex subunit 5	−2.32
Q63768	Adapter molecule crk	−2.32
P26043	Radixin	−2.23
Q63584	Transmembrane protein Tmp21	−2.20
P27274	CD59 glycoprotein	−2.18
Q3T1I4	Protein PRRC1	−2.16
P62142	Serine/threonine-protein phosphatase PP1-beta catalytic subunit	−2.15
P55002	Microfibrillar-associated protein 2	−1.39
Q04857	Collagen alpha-1(VI) chain	−1.36
P04157	Receptor-type tyrosine-protein phosphatase C	−1.22
P11240	Cytochrome c oxidase subunit 5A, mitochondrial	−1.22
P15508	Spectrin beta chain, erythrocyte	−1.20
P31399	ATP synthase subunit d, mitochondrial	−1.15
Q62737	Cytochrome b-245 light chain	−1.10
Q02788	Collagen alpha-2(VI) chain	−1.09
P26051	CD44 antigen	−1.08
Q8BUR4	Dedicator of cytokinesis protein 1	−1.06
P11662	NADH-ubiquinone oxidoreductase chain 2	−1.03
P24623	Alpha-crystallin A chain	−1.03
P61805	Dolichyl-diphosphooligosaccharide--protein glycosyltransferase subunit DAD1	−0.95
P97700	Mitochondrial 2-oxoglutarate/malate carrier protein	−0.90
Q9JM51	Prostaglandin E synthase	−0.88
Q6AXX6	UPF0765 protein C10orf58 homolog	−0.87
P26151	High affinity immunoglobulin gamma Fc receptor I	−0.86
Q9R233	Tapasin	−0.85
Q9CQ91	NADH dehydrogenase [ubiquinone] 1 alpha subcomplex subunit 3	−0.84
P29419	ATP synthase subunit e, mitochondrial	−0.83
Q3B8Q1	Nucleolar RNA helicase 2	−0.83
P06687	Sodium/potassium-transporting ATPase subunit alpha-3	−0.80
P97544	Lipid phosphate phosphohydrolase 3	−0.79
P00697	Lysozyme C-1	−0.77
P11247	Myeloperoxidase	−0.77
P08050	Gap junction alpha-1 protein	−0.77
Q99P82	Claudin-11	−0.77

**Fig. 4:**
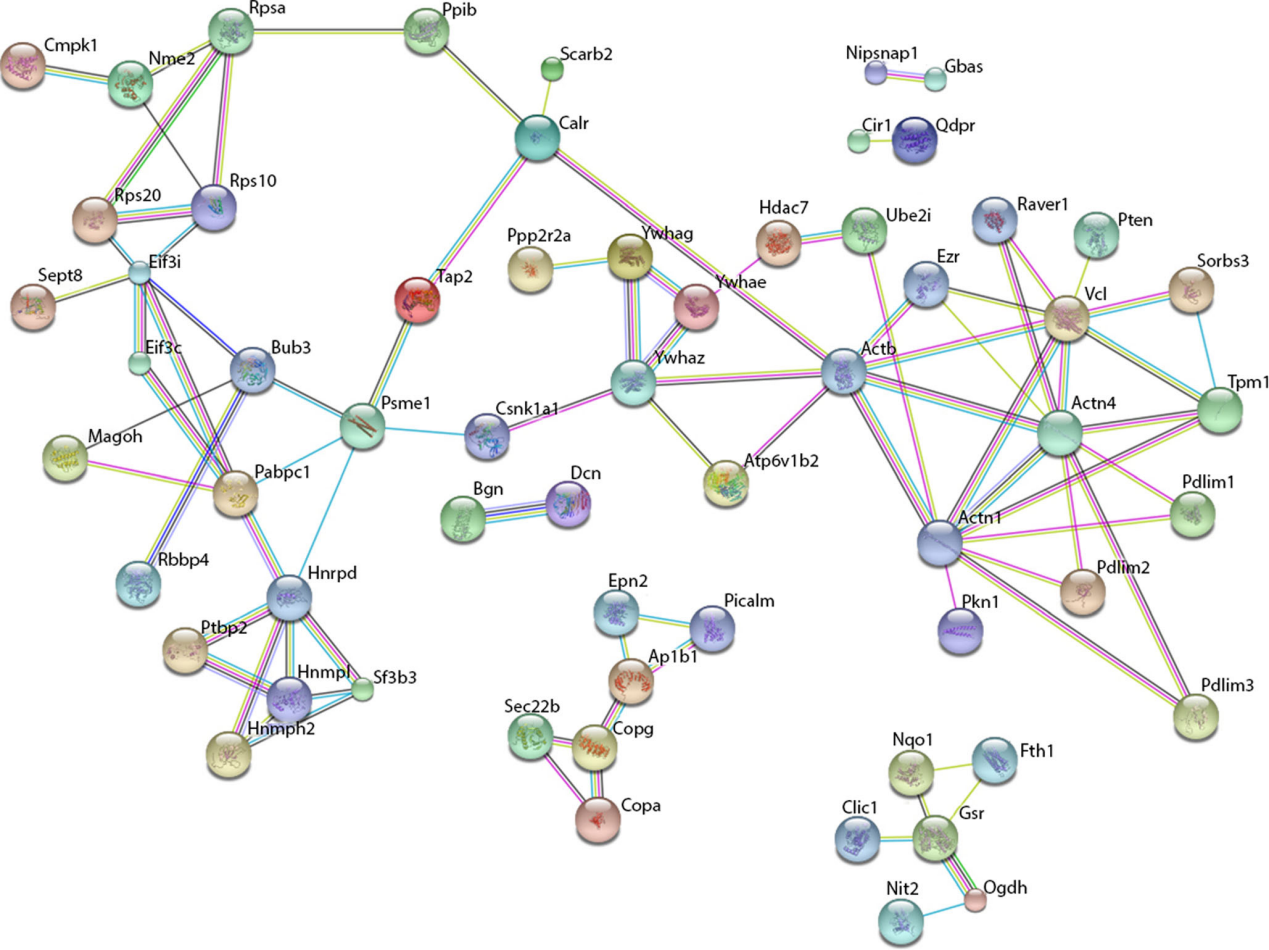
Proteins upregulated after experimental SAH and affected by U0126 treatment (group 1 proteins). Proteins whose upregulation in large cerebral arteries of rats 48 h after SAH did not persist after U0126 treatment were subjected to network analysis performed with STRING (www.string.db) software. The network consists of roughly six clusters, the most predominant containing structural proteins

Network analysis of group 1 proteins was performed using the STRING database for protein interactions^4^ (Figs. 3 and 4). Of the 133 identified proteins, the database recognized 107 and constructed a network of protein interactions (Fig. 4). The analysis showed that many of the proteins were regulated through each other either directly or indirectly. In particular, the analysis revealed a network of proteins with structural functions which includes actinin and vinculin. Actinin is a structural protein and it is involved in cytoskeletal stability. Vinculin is a protein involved in focal adhesions via interaction with actin and other structural proteins (Sun et al. 2008). Another group of highly interconnected proteins includes three isoforms of the adaptor protein 14-3-3 (14-3-3 protein zeta/delta, gamma, and epsilon) (Fig. 6). The 14-3-3 adaptor protein has a large number of reported interaction partners and roles in apoptosis, cell cycle, migration, and differentiation (Mhawech 2005). Networks of proteins involved in translation, mRNA processing, and protein folding such as translational initiation factor 3 subunits, Polyadenylate-binding protein 1 and ribosomal protein subunits S40 isoforms were also identified. Furthermore, a network of proteins responsible for vesicle transport and formation including epsin and phosphatidylinositol-binding clathrin assembly protein and a network of reductases were found including quinone reductase 1 and glutathione reductase.

**Fig. 5:**
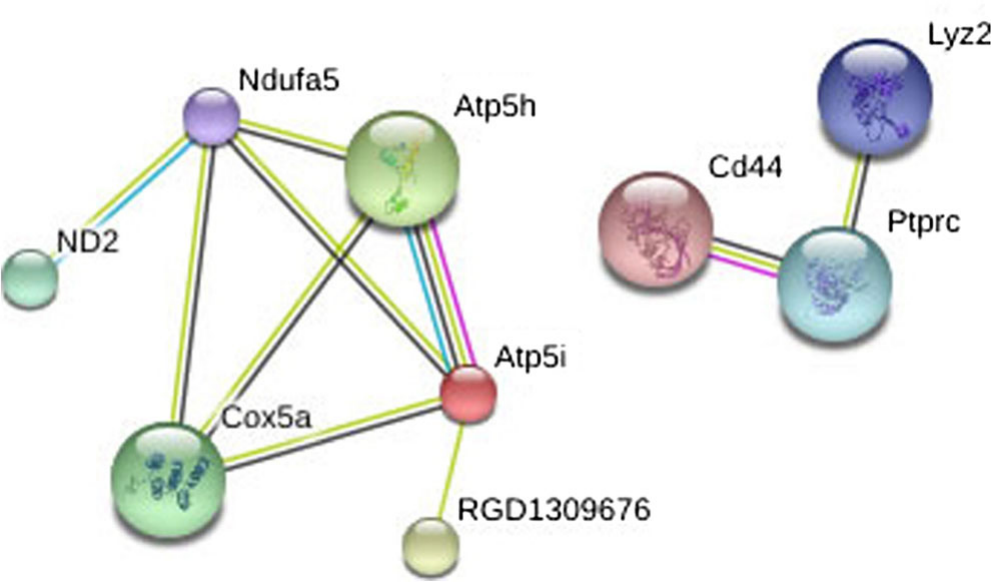
Proteins downregulated in experimental SAH and affected by U0126 treatment (group 2 proteins). Proteins whose downregulation in large cerebral arteries of rats 48 h after SAH did not persist after U0126 treatment were subjected to network analysis performed with STRING (www.string.db) software. The network consists of two individual clusters, the most predominant containing proteins involved in cellular metabolism

When performing a network analysis of those proteins whose SAH-induced downregulation disappeared after U0126 treatment (group 2 proteins), two networks were suggested (Fig. 5). One network included six proteins involved in metabolism such as ATP synthase and cytocrome C synthase. The other network contained three proteins involved in immune responses.

### Validation of Specific Proteins and Their Regulation

**Fig. 6:**
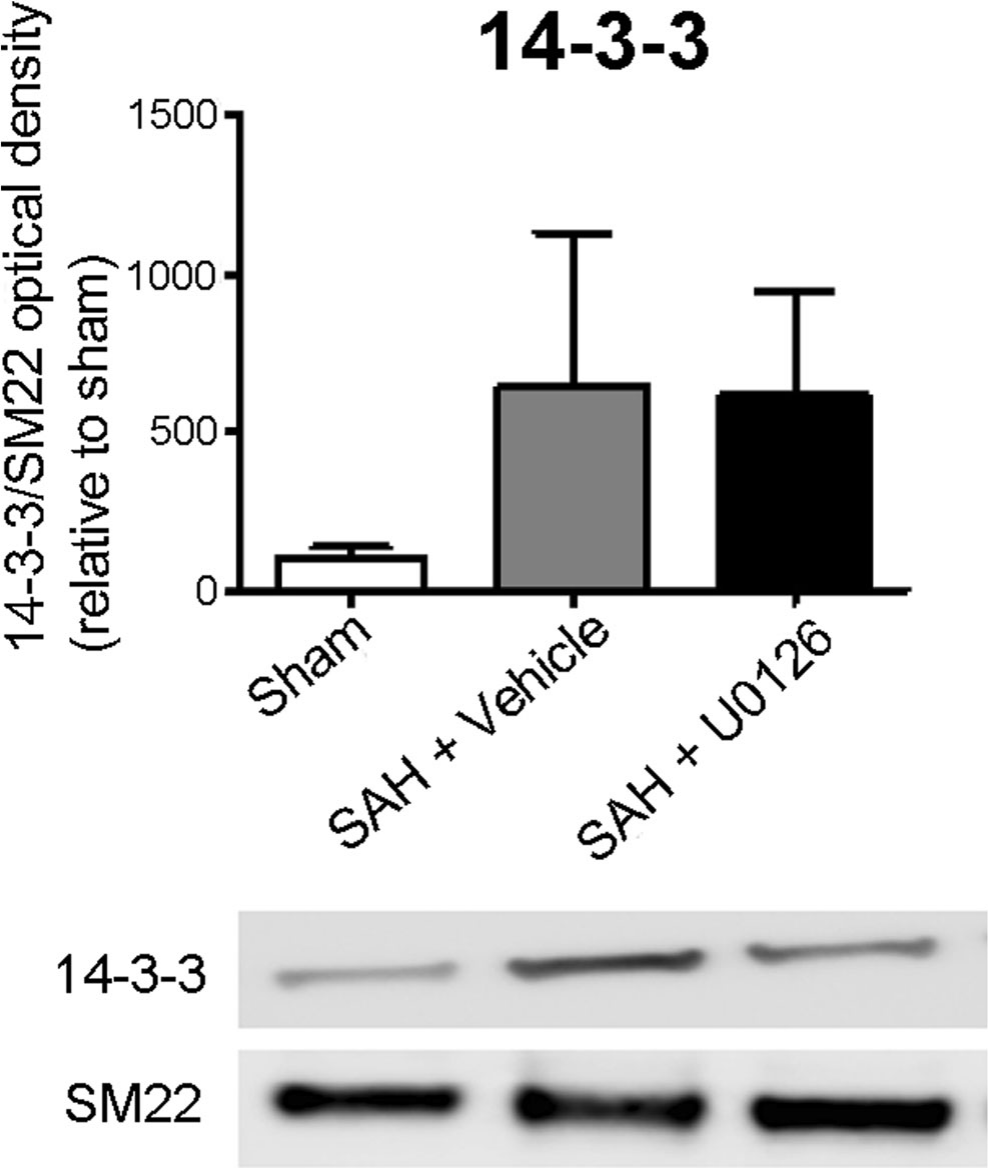
Validation of expression of 14-3-3 in large cerebral arteries. The expression of 14-3-3 in large cerebral arteries of sham animals, and animals subjected to SAH and treated with vehicle or U0126 was investigated by Western blot. 14-3-3 is present in all groups and was upregulated in both the vehicle and the U0126-treated groups (*p* < 0.05). Data were analyzed with one-way ANOVA and Newman-Keuls test for multiple comparisons

We chose to validate proteins from the strong networks of the proteins that were found upregulated after SAH and normalized by MEK1/2 inhibition (group 1 proteins). We studied the expression of 14-3-3 protein in animals subjected to sham surgery or SAH with or without U0126 treatment with Western blot. This protein was detectable in all three groups, supporting that they are indeed expressed in cerebral vessels. Furthermore, the adaptor protein 14-3-3 showed regulation (one-way ANOVA, *p* = 0.05), more specifically 14-3-3 tends to be upregulated after SAH, whereas treatment with U0126 did not affect this upregulation (Fig. 6).

## Discussion

This study is the first to show on an overall proteomic level the expressional changes that occur in large cerebral arteries at 48 h post-SAH and to address how these proteomic changes are affected by treatment with a MEK1/2 inhibitor. Localization studies demonstrated this to occur early after SAH and to maintain elevated levels in the VSMCs (Ansar and Edvinsson 2008). It is worth noting that much of previous work to find novel ways of therapy in order to reduce cerebral vasospasm and the development of late cerebral ischemia after SAH have centered on single molecular approaches. Our study clearly reveals that a large number of proteins show differential expression in the cerebral vessel walls following an experimental SAH and this supports our vision that therapy must be directed towards a common signal transduction mechanism (Edvinsson and Povlsen 2011).

We have shown that the MEK/ERK pathway is activated in cerebral ischemia and that the activation results in enhanced expression of contractile cerebrovascular receptors such as endothelin type B, 5-hydroxytryptamie type 1B, angiotension II type AT1, and thromboxane receptors in the cerebral arteries associated with the ischemic region. Most interesting the specific inhibitor U0126 not only prevents this receptor upregulation but importantly has a beneficial effect on the outcome after experimental subarachnoid hemorrhage in rats (Larsen et al. 2011). Compared to rats treated with vehicle, the U0126-treated rats perform better on a rotating pole test and showed increased spontaneous activity, rearing, grooming, and locomotion as compared to vehicle-treated animals. These findings are in line with and extend previous work (Edvinsson and Povlsen 2011; Larsen et al. 2011; Parker et al. 2013). The present study thus adds to the evidence pointing to the MEK/ERK pathway as a major player in SAH pathophysiology and outcome.

The proteome of major cerebral arteries after SAH revealed that pathways linked to structural modifications of the cerebral blood vessel wall, mainly the VSMCs, are highly regulated after SAH. In addition, we found that proteins involved in binding of nucleic acids, proteins, or calcium ions, and in catalytic activity are highly regulated.

The main results of this study is the identification of 250 proteins regulated in cerebral vessels after SAH and the finding that 184 of these proteins returned to sham level or were contra regulated after treatment with U0126. This underlines the importance of the MEK/ERK pathway in cerebrovascular pathology after SAH. The proteins identified in this study comprise strong networks of structural proteins, adaptor proteins, and in addition networks of proteins involved in translation, mRNA processing, and protein folding.

Remodeling of cerebral arteries is a well-known phenomenon to occur after SAH (Edvinsson and Povlsen 2011; Zhang and Macdonald 2006) and the high number of structural proteins that were observed to be regulated at 2 days after induction of SAH in this study supports that a structural remodeling takes place after SAH. Among the structural proteins we observed to be upregulated by SAH was actinin, an important structural protein in smooth muscle cells. Actinin acts as a linker between integrins and actin filaments to strengthen the cytoskeleton and mediate contraction of the smooth muscle cell (Sun et al. 2008). Another upregulated structural protein is vinculin, a focal adhesion protein involved in binding integrins and actin in the cytoskeleton. Vinculin plays a role in the connection between integrins and actin filaments in the vasculature (Critchley 2004). The network of structural proteins also included tropomyosin and β-actin, both involved in contractility of VSMCs.

Interestingly, the network analysis in addition highlighted the 14-3-3 protein family, whose members previously have been shown to affect the ability of VSMCs to undergo phenotypic switch in vitro (Chen et al. 2013). The 14-3-3 proteins are a family of conserved regulatory molecules that are expressed in all eukaryotic cells, and we validated their presence in cerebral arteries by Western blot. The 14-3-3 proteins have the ability to bind a multitude of functionally diverse signaling proteins, including kinases, phosphatases, and transmembrane receptors. More than 50 signaling proteins have been reported as 14-3-3 ligands including proteins of the Ras/Raf/MEK/ERK pathway (Fischer et al. 2009; Fu et al. 2000).

Upregulation of proteins involved in the protein synthesis pathways of the VSMCs is in line with the fact that in addition to the structural proteins previously mentioned, several proteins such as receptors, cytokines, and ion channels have been observed to be upregulated (Edvinsson and Povlsen 2011; Kamp et al. 2012; Maddahi et al. 2012). Their upregulation might depend severely on a more efficient protein synthesis and thereby an upregulation of the protein synthesis pathways. However, neither the receptors nor the Ca^2+^ channels were identified as regulated in this study, but this might very well be explained by their relatively low abundance compared to, e.g., structural proteins.

The downregulation of mitochondrial proteins is difficult to explain. It seems like a paradox that energy-consuming processes such as protein synthesis are upregulated, while energy-generating pathways are downregulated. However, we speculate that the downregulation is not beneficial since it is abolished after treatment with MEK inhibition, which according to our findings is beneficial after SAH (Fig. 1).

We show that targeting the MEK-ERK1/2 pathway improves outcome of animals and normalizes protein changes 48 h after SAH. Previous work on SAH have focused on limited targets, e.g., single receptors, cytokines, or matrix metalloproteinases (Ansar et al. 2007; Maddahi et al. 2012; Wang et al. 2012); however, our approach made it possible to draw a global picture of multiple changes. One example of the “single target” approach is the Clazosentan trial project (an endothelin receptor antagonist). By targeting the endothelin receptors with a specific antagonist Clazosentan, the arterial narrowing of the vessels was reduced, but it did not improve outcome after SAH (Macdonald et al. 2012). Therefore it seems that additional processes play a role in the vascular pathology after SAH and a total quantitative proteome is a promising tool to get a broader view of the events that follow SAH.

In summary, the overall findings of this study suggest that the changes in structural properties of VSMC change after SAH in a very complex way. These changes can be reduced by treatment with U0126 and this reduction is associated with improved neurological outcome. This suggests that MEK1/2 inhibition after SAH might be a possible treatment strategy for SAH patients. Moreover, the data from this study comprise a large dataset that could be very useful for further investigations and validation of other pathways involved in the complex aftermath of SAH with or without treatment with U0126.

## Electronic Supplementary Material

Fig. S1 (DOC 79 kb)

Fig. S2 (DOC 80 kb)

Table S1 (DOC 49 kb)

Table S2 (DOC 46 kb)

## Footnotes

1 One protein can have several functions and it might therefore appear in more than one group. Additionally, the database did not allocate all the identified proteins.

2 Hits are defined as the total number of molecular functions assigned for the recognized proteins.

3 In the PANTHER classification system, binding is defined as calcium ion binding, calcium-dependent phospholipid binding, chromatin binding, lipid binding, nucleic acid binding, and protein binding (www.pantherdb.org).

4 STRING did not allocate interactions to all identified proteins. The network analyses only contain proteins where interactions could be allocated.
