# Supplementary material for: Proteomic Expression Changes in Large Cerebral Arteries After Experimental Subarachnoid Hemorrhage in Rat Are Regulated by the MEK-ERK1/2 Pathway
Source: J Mol Neurosci. 2017 Jul 24;62(3):380–94. doi: 10.1007/s12031-017-0944-7 (PMC5541124; doi:10.1007/s12031-017-0944-7)
Supplement: Supplementary file 1 — (DOC 79 kb) [file 12031_2017_944_MOESM1_ESM.doc]

**Figure S1**

**6h**

**12h**

**48h**

SAH

**48h**

**24h**

**36h**

**Treatment**

Euthanasia

Euthanasia

**Figure S1:** **Timeline for treatment with U0126.** Rats were subjected to SAH and treated with either U0126 or vehicle (DMSO) at 6 h, 12 h, 24 h and 36 h post insult. At 48 h the rats were sacrificed. Control animals underwent sham operation, received no treatment and were sacrificed 48 h after surgery.
