# Supplementary material for: Proteomic Expression Changes in Large Cerebral Arteries After Experimental Subarachnoid Hemorrhage in Rat Are Regulated by the MEK-ERK1/2 Pathway
Source: J Mol Neurosci. 2017 Jul 24;62(3):380–94. doi: 10.1007/s12031-017-0944-7 (PMC5541124; doi:10.1007/s12031-017-0944-7)
Supplement: Supplementary file 2 — (DOC 80 kb) [file 12031_2017_944_MOESM2_ESM.doc]

**Figure S2**

**Figure S2: Molecular functions of proteins regulated after SAH and treatment with DMSO as compared with sham**. All subarachnoid hemorrhage-regulated proteins were grouped in functional categories according to their molecular function by the PANTHER database (www.panther.db). Blue bars represent upregulated proteins and red bars represent downregulated. Proteins with functions of catalytic activity, binding and structural activity represents the largest groups of both up- and downregulated proteins.
