# Supplementary material for: Proteomic Expression Changes in Large Cerebral Arteries After Experimental Subarachnoid Hemorrhage in Rat Are Regulated by the MEK-ERK1/2 Pathway
Source: J Mol Neurosci. 2017 Jul 24;62(3):380–94. doi: 10.1007/s12031-017-0944-7 (PMC5541124; doi:10.1007/s12031-017-0944-7)
Supplement: Supplementary file 3 — (DOC 49 kb) [file 12031_2017_944_MOESM3_ESM.doc]

**Table S1**

| ***A: Name of primary antibody*** | ***Manufacturer*** | ***Dilution*** |
| --- | --- | --- |
| Monoclonal anti-vinculin antibody (V9131) | **Sigma** | 1:1000 |
| Polyclonal α-Actinin antibody (#3134) | Cell signaling | 1:1000 |
| Polyclonal anti 14-3-3 antibody (ab9063) | Abcam | 1:1000 |
| Monoclonal anti-GAPDH antibody (D16H11) | Cell signaling | 1:10.000 |
| Polyclonal anti-SM22α antibody (ab10135) | Abcam | 1:10.000 |

| ***B: Name of secondary antibody*** | ***Manufacturer*** | ***Dilution*** |
| --- | --- | --- |
| Anti-mouse IgG (NA931V) | GE Healthcare | 1:10.000 |
| Anti-rabbit IgG (NA934V) | Ge Healthcare | 1:10.000 |
| Anti-goat IgG (#31433) | Pierce | 1:10.000 |

**Table S1: Primary and secondary antibodies used for Western blotting**

The table shows primary and secondary antibodies used for Western blotting and their dilutions
