# Supplementary material for: Proteomic Expression Changes in Large Cerebral Arteries After Experimental Subarachnoid Hemorrhage in Rat Are Regulated by the MEK-ERK1/2 Pathway
Source: J Mol Neurosci. 2017 Jul 24;62(3):380–94. doi: 10.1007/s12031-017-0944-7 (PMC5541124; doi:10.1007/s12031-017-0944-7)
Supplement: Supplementary file 4 — (DOC 46 kb) [file 12031_2017_944_MOESM4_ESM.doc]

**Table S2**

| ***Parameter*** | ***Baseline*** | ***After blood injection*** |
| --- | --- | --- |
| MABP | 88 ± 13 mmHg | 89 ± 15 mmHg |
| ICP | 6 ± 2 mmHg | 106 ± 12 mmHg |
| Cortical flow | 100% | 20 ± 4% |

**Table S2: Physiological parameters during experimental SAH**

The table shows the physiological parameters monitored during experimental SAH in rat before and after injection of blood. Compared to baseline (level before injection) the MABP remained unchanged, the ICP was elevated above MABP and the cortical blood flow was reduced to 20 ± 4% of baseline.
